# Stage 4 Takayasu Retinopathy With Persistent Neovascularization

**DOI:** 10.7759/cureus.16640

**Published:** 2021-07-26

**Authors:** Siti Amra Abd Rahman, Safinaz Mohd Khialdin, Rosiah Muda

**Affiliations:** 1 Ophthalmology, Hospital Universiti Kebangsaan Malaysia, Kuala Lumpur, MYS; 2 Ophthalmology, Hospital Sultanah Nur Zahirah, Kuala Terengganu, MYS

**Keywords:** takayasu arteritis, resistant, neovascularization, hypoperfusion, immunosuppressive agents

## Abstract

Takayasu arteritis is a chronic, progressive, autoimmune, granulomatous, medium-to-large vessel-panarteritis. It may cause chronic ocular ischaemia that may be refractory to treatment. We report a case of stage 4 Takayasu retinopathy resistant to conventional treatments. The patient was a 22-year-old woman who was diagnosed with Takayasu arteritis when she first presented with claudication while chewing and swallowing for one month. The patient was found to be hypertensive, with a significant systolic blood pressure difference between the arms and non-palpable bilateral brachial and radial artery pulses. Angiogram imaging revealed abnormalities involving the left subclavian, bilateral common carotid and left internal carotid arteries.

She was referred to the ophthalmology clinic, as she experienced bilateral recurrent transient visual loss six months after the diagnosis. Dilated fundus examination showed bilateral stage 2 Takayasu retinopathy, evidenced by the presence of dilated retinal veins with microaneurysms. Her eyes progressed to stage 4 Takayasu retinopathy with proliferative retinopathy within one year of immunomodulatory therapy, largely due to poor compliance. No signs of regression were observed after completion of bilateral pan-retinal photocoagulation (PRP) with ongoing immunosuppressive treatment.

## Introduction

Takayasu arteritis is a rare, idiopathic, autoimmune, progressive, chronic, granulomatous vasculitis, affecting medium- and large-sized arteries, leading to multi-organ damage [1].

Ninety percent of cases involve the aorta, subclavian and carotid arteries; meanwhile, 37% of cases develop Takayasu retinopathy throughout the disease course [2]. Approximately, 20% of Takayasu arteritis cases are resistant to therapy, and despite compliance to immunosuppressive therapy, remission is only seen in half of the treated cases [1].

We report a case of stage 4 Takayasu retinopathy that was resistant to conventional treatments.

## Case presentation

A 22-year-old Malay woman came to the emergency department with intermittent claudication while chewing and swallowing for one month. Physical examination showed the absence of bilateral radial and brachial artery pulses. The right carotid artery pulse was palpable with a bruit on auscultation, while the left carotid artery pulse was non-palpable. There was a significant difference in systolic blood pressure between the right and left arms of 10 mmHg and a raised bilateral popliteal blood pressure of 180/110 mmHg.

Her erythrocyte sedimentation rate was high (116 mm/hour). The magnetic resonance angiogram (MRA) imaging study reported right common carotid artery stenosis with total occlusion of the left subclavian, left common carotid, and left internal carotid arteries; meanwhile, the aortic arch, brachiocephalic trunk, and thoracic and abdominal aorta were spared. She was diagnosed with Takayasu arteritis and started immunosuppression therapy with oral prednisolone and intravenous cyclophosphamide.

**Figure 1:**
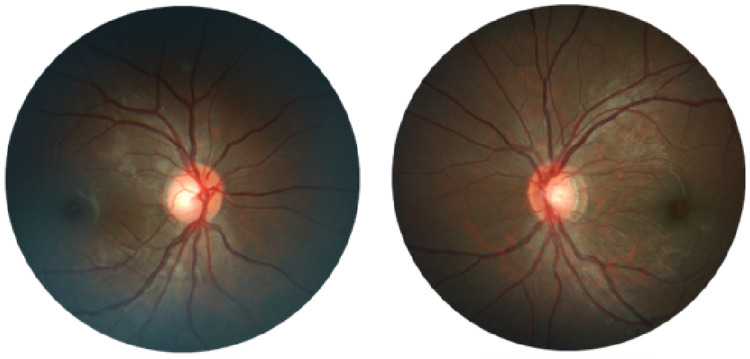
Fundus photos show dilated retinal veins with microaneurysms

She was referred to our Ophthalmology Department for bilateral recurrent transient vision loss that started six months after the diagnosis. At the initial presentation, her best-corrected visual acuity was 6/9 in the right eye and 6/36 in the left. Anterior segment and intraocular pressure were normal in both eyes. Fundus showed dilated retinal veins with multiple microaneurysms (Figure 1).

**Figure 2:**
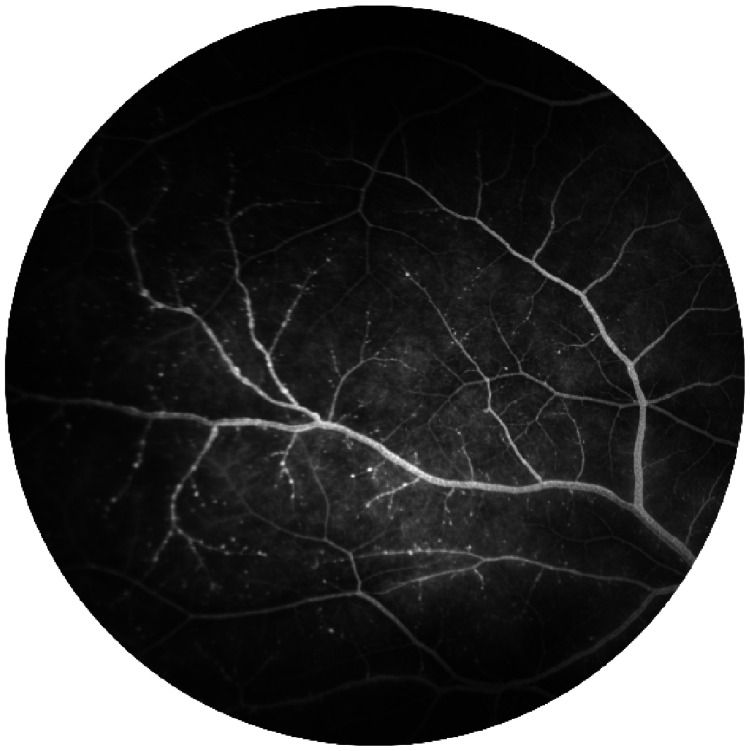
Right eye fundus fluorescein angiography shows microaneurysms at superotemporal quadrant

**Figure 3:**
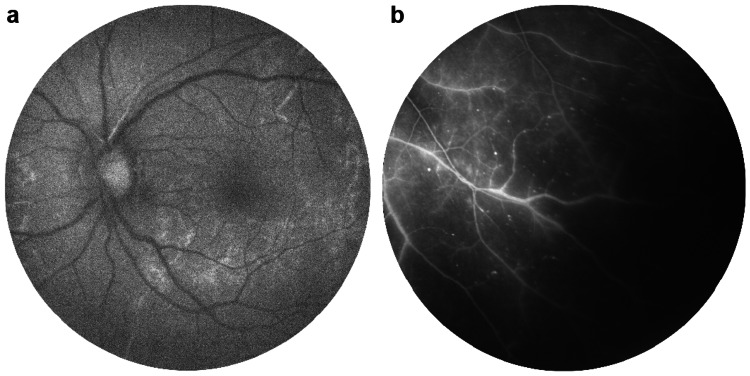
Left eye fundus fluorescein angiography shows prolonged arm-retina time, 18 seconds (a); left eye fundus fluorescein angiography shows the area of capillary fall-out at inferotemporal quadrant (b)

Results from fundus fluorescein angiography (FFA) were normal in the right eye, except for a few microaneurysms (Figure 2). The left eye, however, showed prolonged arm-retina time, delayed arteriovenous (AV) filling and staining of the vein wall with areas of capillary drop-out inferotemporally (Figure 3a and 3b). Retinal photocoagulation was performed in the ischaemic retina.

**Figure 4:**
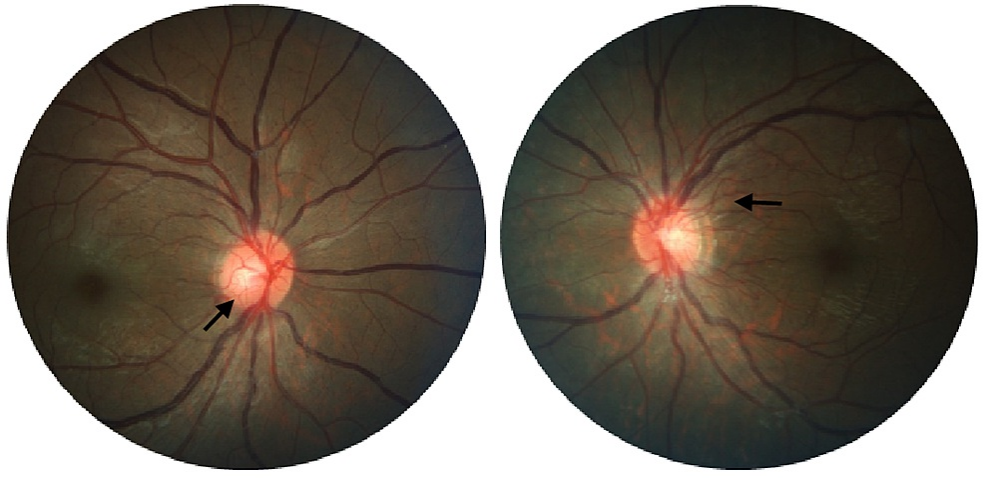
Fundus photos show dilated and tortuous retinal veins with new vessels at optic discs (arrows)

**Figure 5:**
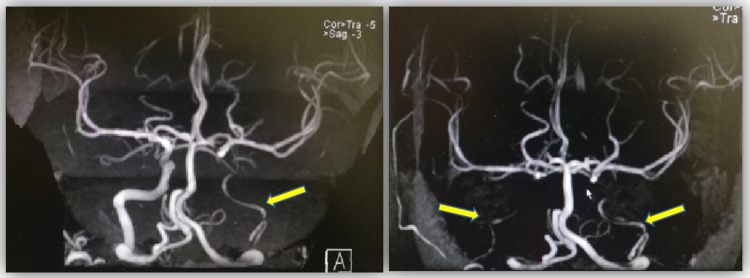
Initial MRA shows minimal flow signal in the left internal carotid artery (arrow) (a); repeated MRA over a year shows complete loss of flow signals in the right and left internal carotid artery (arrows) (b) MRA: magnetic resonance angiography.

However, despite six cycles of intravenous cyclophosphamide, immunosuppressive agents (azathioprine and methotrexate), and oral prednisolone, administered for over a year, the disease progressed to full-blown bilateral hypoperfusion retinopathy with florid neovascularization due to poor compliance (Figure 4). Magnetic resonance imaging (MRI) angiography were repeated, and they revealed a complete loss of flow signal in the right common and internal carotid arteries (Figure 5a and 5b), with new brain infarction.

**Figure 6:**
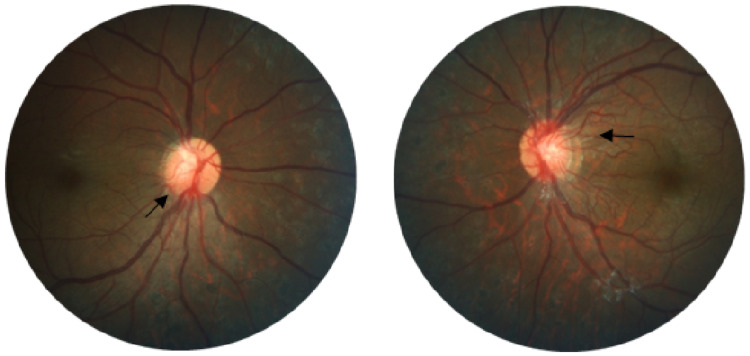
Fundus photos show persistent neovascularisations (arrows) at optic discs with pan-retinal photocoagulation laser scars

The patient subsequently underwent bilateral pan-retinal photocoagulation (PRP) and the rheumatologist commenced treatment with oral mycophenolate mofetil. Even though the patient’s visual acuity remained stable, neovascularization at the discs (NVD) persisted, showing no signs of regression (Figure 6).

## Discussion

Takayasu arteritis causes attenuation and obliteration of branches of the aorta, leading to cerebral and ocular ischaemia [3]. There are few diagnostic criteria for Takayasu arteritis. The two most common are the Ishikawa criteria 1988 and the American College of Rheumatology (ACR) criteria 1990 [4,5]. Based on Ishikawa criteria, the patient met one major criterion (occlusion of the left subclavian artery) and three minor criteria (hypertension with high ESR and occlusion of the left common carotid artery).

Another widely used diagnostic criteria of Takayasu arteritis are the ACR criteria [5]. Apart from similar age criteria (40 years old or younger), others include claudication of extremities, decreased brachial artery pulse, a systolic blood pressure difference of at least 10 mmHg between the two arms, bruits over subclavian arteries or aorta, and focal or segmental arteriographic narrowing or occlusion of the aorta or its primary branches or large arteries which is not due to arteriosclerosis or fibromuscular dysplasia [5]. The presence of at least three criteria is a strongly suggestive of Takayasu arteritis, with a high sensitivity of 90.5% and a specificity of 97.8% [5]. These criteria allow greater flexibility and hence variability in actual clinical practice and help prevent the possibility of misdiagnosis. This patient fulfilled almost all the ACR diagnostic criteria: young age, claudication of mastication and laryngeal muscles supplied by carotid arteries, non-palpable brachial artery pulses, a difference in systolic blood pressure of 10 mmHg between the two arms, and arteriogram abnormalities involving the left subclavian, bilateral common carotid and left internal carotid arteries.

The most common ocular manifestations include Ocular Ischaemic Syndrome and Anterior Ischaemic Optic Neuropathy (AION) [1]. Ischaemic ocular manifestation is best described as Takayasu retinopathy, further classified into four stages by Uyama and Asayama: distensions of retinal veins, micro-aneurysm, arteriovenous anastomoses, and ocular complications such as cataract, rubeosis iridis, retinal ischaemia, neovascularization, and vitreous haemorrhage [3]. As seen in this case, the patient first presented with dilated and tortuous retinal veins and multiple microaneurysms, bilaterally, which was stage 2, and progressed to neovascularization, stage 4, within a year.

While managing microaneurysms and neovascularisations, the aim is to suppress the inflammatory process and preserve vascular capability [1]. The administration of systemic steroids, methotrexate as an immunomodulator, and aspirin is advocated [1]. Additionally, Leflunomide, a tumour necrosis factor (TNF) α antagonist, and biologic agents, Tocilizumab and Ustekinumab, have also shown promising effects in treating refractory Takayasu arteritis [6,7]. Laser PRP is also used to ablate the ischaemic peripheral retina [3]. Due to poor adherence to medications and follow-ups, the patient’s disease progressed, and neovascularization persisted despite intensive laser PRP. In a recent report, intravitreal injection of anti-vascular endothelial growth factor antibody (anti-VEGF) effectively achieved complete regression of neovascularization and improved visual outcomes [8]. Thus, anti-VEGF might be considered for treating the patient in the present study.

Fludeoxyglucose positron emission tomography (FDG-PET) scan is a new diagnostic tool that can detect vascular inflammatory lesions before structural abnormality develops [7]. It has been reported to be safe in renal insufficiency and has high sensitivity and specificity with concurrent usage of CT scans [7]. Thus, this patient is scheduled for FDG-PET in a few months in order to scan her entire vascular tree to look for vasculitic lesions.

Endovascular or vascular revascularization may be considered in this patient to reverse the complications, although such approaches have variable rates of restenosis [6].

The visual outcomes of Takayasu retinopathy depend on three factors: the portions of carotid arteries affected, the rate of development and duration of retinal vascular insufficiency, and the effectiveness of collateral ocular blood supply [1]. In this case, close and regular follow-ups are mandatory to monitor ocular complications, such as vitreous haemorrhage, retinal detachment, and neovascular glaucoma.

## Conclusions

Takayasu arteritis imposes a great challenge to ophthalmologists despite being a rare cause of retinopathy. Multidisciplinary collaboration is mandatory to achieve the best therapeutic approach in this systemic disease. Patient’s motivation and compliance to treatments and follow-ups are important factors that could prevent further deterioration of the disease. Biologic therapy and surgical interventions may improve the prognosis of this disease. Besides, intravitreal injection of anti-VEGF can be the treatment next-in-line for persistent neovascularization.
